# Faces and words are both associated and dissociated as evidenced by visual problems in dyslexia

**DOI:** 10.1038/s41598-021-02440-7

**Published:** 2021-11-26

**Authors:** Heida Maria Sigurdardottir, Alexandra Arnardottir, Eydis Thuridur Halldorsdottir

**Affiliations:** Icelandic Vision Lab, Department of Psychology, University of Iceland, Saemundargata 12, 102 Reykjavik, Iceland

**Keywords:** Psychology, Human behaviour, Cognitive neuroscience, Dyslexia, Perception

## Abstract

Faces and words are traditionally assumed to be independently processed. Dyslexia is also traditionally thought to be a non-visual deficit. Counter to both ideas, face perception deficits in dyslexia have been reported. Others report no such deficits. We sought to resolve this discrepancy. 60 adults participated in the study (24 dyslexic, 36 typical readers). Feature-based processing and configural or global form processing of faces was measured with a face matching task. Opposite laterality effects in these tasks, dependent on left–right orientation of faces, supported that they tapped into separable visual mechanisms. Dyslexic readers tended to be poorer than typical readers at feature-based face matching while no differences were found for global form face matching. We conclude that word and face perception are associated when the latter requires the processing of visual features of a face, while processing the global form of faces apparently shares minimal—if any—resources with visual word processing. The current results indicate that visual word and face processing are both associated and dissociated—but this depends on what visual mechanisms are task-relevant. We suggest that reading deficits could stem from multiple factors, and that one such factor is a problem with feature-based processing of visual objects.

## Introduction

The same object can project an almost infinite number of images onto our retina. It can be seen from afar or near, from the left or right, from the top or bottom, occluded by other objects, in different backgrounds, in bright sunshine or twilight. When compared pixel-by-pixel, such images might have less in common than images of two completely different objects, such as two people seen from the same viewpoint or two words such as CAT vs. OAT (compare to *cat* and *oat*). These challenges are often collectively grouped under the term high-level vision and are generally thought to be solved by later stages of the ventral visual stream^1^. The ventral stream supports the visual perception and recognition of complex forms and objects^2–6^, including visually presented faces and words.

Several studies have focused on the domain-generality or domain-specificity of visual word and face processing, both behaviorally and in terms of their neural substrates in the ventral stream (e.g.^7–24^). On the surface, faces and words have little in common. Accordingly, as noted by Robotham and Starrfelt^21^, the double dissociation of word and face processing is textbook knowledge. Farah famously reviewed dozens of case studies on people with visual associative agnosias for words, faces, or other objects^12,25,26^. The patterns of co-occurrences among these agnosias were consistent with the existence of two underlying visual recognition abilities, one highly important for words and not needed for faces, and another of high importance for faces and not needed for words^26^. Faces and words also consistently evoke high activity in relatively anatomically separable neural patches of the high-level ventral stream^2^, often interpreted in support of their domain-specificity.

However, links between face and word processing have more recently been proposed. For example, Dehaene et al.^11^ suggested that literacy, like other forms of expertise, leads to cortical competition effects in regions of the ventral visual stream (see also^27,28^). More specifically, literacy seems to induce competition with the representation of faces in the left fusiform gyrus, leading the authors to speculate that our face perception abilities suffer in proportion to our reading skills. Behrmann and Plaut^7,29^ also offer an alternative to the traditional view that higher levels of the ventral visual stream consist of independent domain-specific regions dedicated to the processing of particular categories. They acknowledge that faces and words have the strongest claim of all object classes to domain-specificity, with the potential for distinct cortical regions specialized for their high-level visuoperceptual analysis. They however argue that face and word representations are not independent, and that functional specialization of brain regions is graded, and cite the partial co-mingling of face and word processing, the association between the acquisition of word and face recognition skills, and their related neural mechanisms. In a critical response to Behrmann and Plaut, Susilo and Duchaine^23^ suggest that at least some of the mechanisms involved in face and word processing are independent and cite neuropsychological cases showing a double dissociation between face and word recognition (see also Behrmann and Plaut^7^ for their response to Susilo and Duchaine^23^). Robotham and Starrfelt^21^ also provide convincing evidence that face and word recognition abilities can be selectively affected.

An almost entirely independent large body of work concerns the possible causes of developmental dyslexia, and visual factors are not generally thought to play a role (but see e.g.^30,31^; for reviews, see e.g.^32,33^). However, according to our new high-level visual dysfunction hypothesis, reading problems could in some cases be a salient manifestation of a deficit of visual cognition stemming from disrupted functioning within the ventral visual stream. For a recent review on this hypothesis, where we discuss work on functional neuroimaging, structural imaging, electrophysiology, and behavior that provides evidence for a link between high-level visual impairment and dyslexia, see^34^. Supporting the hypothesis, hypoactivity of ventral stream areas, particularly in the left hemisphere, appears to be a universal marker for dyslexia as it is found both for dyslexic children and adults, and across deep and shallow orthographies^35–37^). As hypoactivation in the left fusiform and bilateral occipitotemporal gyri is already present in preliterate children with a familial risk for dyslexia^38^ (see also^39^), functional abnormalities of ventral stream regions are unlikely to reflect only reading failure, might not be specific to print, and may play a causal role in dyslexia.

Accordingly, our previous studies indicate that some people with dyslexia have problems with tasks thought to rely on high-level ventral stream regions, including the visual perception and recognition of faces^40–43^. Studies on the face perception abilities of dyslexic readers are however quite inconsistent, with some studies reporting abnormalities^14,39,41–48^, other studies find no such thing^49–53^ and yet other studies are mixed^54,55^ (for details, see^41^). A possible reason for this discrepancy is that it could be the wrong question to ask whether faces and words are associated or dissociated, as the answer could be: Both and neither, depending on what visual characteristics or neural mechanisms are important for the task at hand.

What types of visual characteristics and neural mechanisms might these be? As regions hypoactive in dyslexic readers^37^ may overlap with face-selective regions of the left ventral visual stream^43^, a starting point is to briefly go over known characteristics of visual face processing in the left hemisphere (for a review on laterality effects in face perception, see^56^). While right hemisphere regions appear to be automatically recruited by faces, left hemisphere regions seem to be flexibly recruited based on context, task, or attentional demands^57–59^. Left hemisphere face processing is however not just a poor replica of that of the right, as it excels in some types of face analysis. The left hemisphere shows an advantage in a same-different task for faces when the faces can only be distinguished based on a feature (e.g., different nose^60–62^). Later neuroimaging studies have also indicated that left hemisphere regions are relatively more involved in part- or feature-based face processing while the right hemisphere regions are more important for processing whole faces^63^.

This is a particularly interesting pattern, as configural (or holistic/global) and feature-based processing might provide a dual route to recognition (^12,64^; concept use varies^64–66^ but configural processing is sometimes used interchangably with holistic or global processing, and feature-based processing is sometimes referred to as featural, componential, part-based, local, or analytical processing). Although holistic or configural processing of words contributes to reading to a degree, recognition by parts is generally thought to be of much greater importance^67–70^ (results on featural vs. configural word processing deficits in dyslexic readers are mixed, see e.g.^71,72^). As an anecdotal example, changing a single feature (assuming that letters are features) in the word CAT to BAT will lead to identity changes, while changing the distance between features from CAT to C A T—a global or configural change—will preserve the word’s identity. Holistic processing of faces appears to be intact in dyslexic readers^14,43,71^, as evidenced by normal face inversion and composite face effects, leading us to suggest that they may instead be “…specifically impaired at the part-based [i.e. feature-based] processing of words, faces, and other objects, consistent with their primarily left-lateralized dysfunction of the fusiform gyrus.”^43^. This would be expected to have serious consequences for visual word recognition and less severe yet detectable consequences for other visual tasks that partially rely on such processing. This prediction is tested here.

In the current study, adults with varying degrees of reading abilities, ranging from expert readers to severely impaired dyslexic readers, completed both a feature-based and a global form face matching task. We predicted a dissociation between word and face processing in cases where a face task could effectively be solved by processing the global form of faces (minimal part decomposition), whereas we expected to see an association when a task could most effectively be solved by additionally or instead relying on the feature-based processing of faces (extensive part decomposition). Establishing the association of reading problems with one type of face processing (feature-based) but their dissociation from another type of face processing (global form) provides important information on domain-specificity vs. domain-generality of visual word and face processing and for our high-level visual dysfunction hypothesis of developmental dyslexia.

## Method

### Participants

The stopping rule for data collection was to test a minimum of 60 people but to stop at 80 people if this number was reached within a particular period. A total of 60 people (48 females, 12 males) aged 19 to 51 years (mean 25 years) took part in the study. All were undergraduate students or had graduated less than two years ago. They had Icelandic as their native language and reported normal or corrected vision. As detailed in section Results: Reading Measures, the sample was further subdivided into 24 dyslexic readers (reported previous formal diagnosis of dyslexia or screened positive for dyslexia on the Adult Reading History Questionnaire) and 36 typical readers. The sample was not randomly selected and people with reading problems (diagnosed and undiagnosed) were likely overrepresented. Participants could sign up for a lottery where six participants received a gift certificate for 10.000 ISK.

### Procedure

The study was approved by the National Bioethics Committee of Iceland (protocol 14-027) and reported to the Icelandic Data Protection Authority. The study was performed in accordance with the Declaration of Helsinki and Icelandic guidelines/regulations on scientific studies. Participants were tested in a sound-attenuated chamber. All participants gave informed consent. All tasks were computerized (Dell OptiPlex 760 computer, 17-inch monitor, 1024 × 768 pixels, 85 Hz) using PsychoPy^73,74^. Participants filled out questionnaires on background variables, their history of reading problems, and current and childhood symptoms of ADHD. Participants then completed face perception tasks followed by visual search tasks; viewing distance was set to 57 cm by the use of a chinrest. Participants completed a lexical decision task and were finally asked to read out loud. Data from visual search and lexical decision are analyzed in detail elsewhere^75^ (see also Supplementary Material S4. Regression Models Accounting for Visual Search).

#### Adult Reading History Questionnaire

The Adult Reading History Questionnaire (ARHQ) is a 23-item self-report questionnaire designed to measure people’s history of reading problems^76^ (e.g. “Which of the following most nearly describes your attitude toward school when you were a child?”, “How much difficulty did you have learning to read in elementary school?”). Questions are answered on a 5-point Likert scale ranging from 0 to 4. In this study, the Icelandic version of the ARHQ (ARHQ-Ice^77^) was used. As recommended^77^, only 22 items were analyzed in the current study, resulting in a raw score between 0 and 88; these were rescaled to range from 0 to 1. Higher scores are associated with greater reading difficulties, and a score above 0.43 is a suggested cut-off point when screening for dyslexia^77^. The Icelandic adaptation of the ARHQ is a valid and reliable (Cronbach’s alpha 0.92) screening instrument for dyslexia^77^.

#### Behavioral Evaluation Questionnaire for Adults I and II

Two separate questionnaires regarding ADHD symptoms as defined by the DSM-IV were administered^79^ (e.g. “Fails to give close attention to details or makes careless mistakes in work or schoolwork”, “Fidgets with hands or feet or squirms in seat”). Questionnaires were self-reports of behavior in the past six months (ADHD-I) and childhood symptoms from ages 5 to 12 years (ADHD-II). Participants answered on a 4-point Likert scale, resulting in a total score from 0 to 54 on each list, where higher scores imply more ADHD-related symptoms. These questionnaires are reliable and valid screening tools for ADHD^79^.

#### Face matching

**Figure 1:**
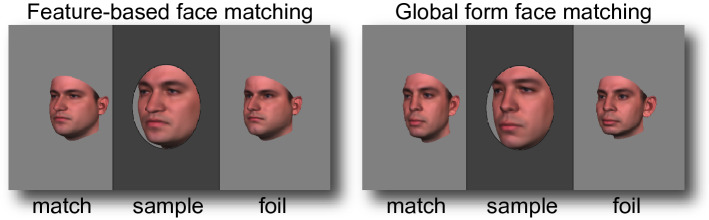
A zoom-in on face stimuli in two face matching trials. A sample face appeared with two choice faces—one foil and one match. The task was to pick the choice face that most resembled the sample face. In feature-based face matching (example on left), the match shared features with the sample. In global form face matching (example on right), the match shared global form with the sample. The foil shared neither features nor global form with the sample. On each trial, all faces looked left (as shown), straight ahead, or to the right, and the match could be on the left (as shown) or right of screen center. Face stimuli are from Van Belle et al.^78^. See https://ppw.kuleuven.be/en/research/lep/resources/face for more examples.

The stimulus set, developed by Van Belle and colleagues^78^, has been used to measure global or configural as well as feature-based processing of faces. As described in Van Belle et al.^78^, the stimulus set was developed from 15 pairs of Caucasian male faces, all with identical skin structure and color and no extra-facial cues (e.g. hair, clothing, or makeup). From each pair of faces, A and B, two new faces were created, one of which had the global form (the form of the skull, muscles and fat structure) of face A and the internal features (e.g. the eyes, nose, and mouth) of face B, and the other whose global form was taken from face B but whose internal features came from face A (see Fig. 1). This resulted in a total of 60 face stimuli. For every face in the stimulus set there was thus one face that differed from it only by its global form and another face that differed from it exclusively by its features.

A trial started with the appearance of a dark gray bar on a light gray background. The bar reached all the way from the top to the bottom of the screen (width approximately 6°). A light gray oval hole or window (approximately 3° × 4°) was shown in the middle of the bar. After 1000 ms, a sample face was shown in the middle of the hole along with two choice faces (match and foil) approximately 5° to the left and right of screen center (Fig. 1). Choice face size was approximately 70% of sample face size. The participant’s task was to determine which of the choice faces was more similar to the sample face. Size differences and the oval window were introduced to minimize possible usage of low-level image-based matching, and to keep accuracy off ceiling. The stimuli stayed onscreen until response. The participants pushed the left arrow key to indicate that the face on the left resembled the sample face, and the right arrow key if the face on the right was deemed more similar to the sample face.

Participants listened to prerecorded instructions, completed two practice trials with cartoon faces, and then completed six blocks of experimental trials with 60 trials per block, 360 trials in total. Global form and feature-based trials were intermixed within blocks. All three faces (sample, match, and foil) had the same orientation in each trial. Trial type (feature-based or global form face matching), orientation (facing 30° left, straight ahead, or 30° right), and location of the match face (left or right of screen center) were fully crossed (30 trials of each combination). Trials appeared in the same randomized order for each subject. Trial order was randomized until there was no correlation between trial order and trial type (R^2^ = 8E−05).

#### IS-FORM and IS-PSEUDO reading tests

Poor readers may compensate for a deficit in a lower-level process, such as word recognition/decoding, by increasingly relying on context^80^. For this reason, the reading tests used are context-free by design. In IS-FORM, participants cannot guess the next to-be-read word based on previously read words, nor can they easily guess the entire word after having read its first few letters as Icelandic is an inflected language so the same word can have many endings (e.g. the word for “reading” can be “lestur”, “lestri”, “lestrar”, “lesturinn”, “lestrinum” etc. depending on context). IS-PSEUDO only includes phonologically valid nonsense words, which by definition do not mean anything, yet dyslexic readers have problems in reading such pseudowords^81,82^.

IS-FORM and IS-PSEUDO reading tests measure (pseudo)words read per minute and percentage of correctly read (pseudo)word forms^40,43^. Dyslexic readers’ performance on both tests has been shown to be markedly poorer than that of typical readers^40,41,43^. IS-FORM includes two lists of 128 words each. One contains common Icelandic word forms and the other uncommon word forms. IS-PSEUDO contains one list of 128 pseudowords. The participants were instructed to read each word list aloud as fast as possible, while making as few errors as possible, in the following order: IS-FORM common, IS-FORM uncommon, and IS-PSEUDO.

### Data analysis and exclusion/inclusion

For the analysis of reaction times, incorrect trials were first removed, and trials with response times ± 3 SDs from an individual’s mean were then excluded for each participant. An alpha level of 0.05 was used for statistical tests, which were all two-sided. Degrees of freedom in reported t-tests were adjusted if Levene’s test for equality of variances was significant. Due to recording failure, two participants had missing values for reading speed and accuracy for the IS-FORM list of common word forms, one had missing values for the IS-FORM list of uncommon word forms, and one had missing values for the IS-PSEUDO list. Reading speed and accuracy values for a missing list were imputed from the reading speed and accuracy of the two other lists using linear regression. Average reading speed (words per minute) and average reading accuracy (percent of correctly read words) were then calculated across the three reading lists for each participant.

## Results

### Reading measures

Our primary reading measures of interest were scores on the Adult Reading History Questionnaire (ARHQ), reading speed, and reading accuracy. Twenty-three people screened positive for dyslexia (ARHQ score of 0.43 or higher), thereof 10 out of 11 people who reported a previous diagnosis of dyslexia. As the 11^th^ person just missed the cutoff (0.43) with an ARHQ score of 0.42 and reported a previous formal diagnosis, this person was included in a group of 24 assumed dyslexic readers. The remaining 36 people were assumed to be typical readers. The two groups will from now on be referred to as dyslexic and typical readers. The groups were well matched in mean age (25 years in both cases) and gender ratios (dyslexic readers: 20.8% males; typical readers: 19.4% males). Dyslexic readers tended to read more slowly and less accurately than typical readers (reading speed: dyslexic readers M = 61 words/minute, *SD* = 14.8; typical readers 82 words/minute, *SD* = 15.6; *t*(58) = 5.430, *p* < 0.001, *d* = 1.440; reading accuracy: dyslexic readers *M* = 90.6%, *SD* = 6.77; typical readers *M* = 96.5%, *SD* = 2.38; *t*(26.833) = 4.078, *p* < 0.001, *d* = 1.163).

### ADHD Measures

Dyslexia and ADHD are highly comorbid^83^. Unsurprisingly therefore, two dyslexic readers but no typical readers reported a previous formal diagnosis of ADHD, and dyslexic readers on average reported greater symptoms of current and childhood ADHD as measured by the Behavioral Evaluation Questionnaires for Adults I and II (ADHD-I: dyslexic readers *M* = 16.6, *SD* = 7.73; typical readers *M* = 8.8, *SD* = 5.29; *t*(58) = 4.674, *p* < 0.001, *d* = 1.185; ADHD-II: dyslexic readers M = 22.5, *SD* = 13.07; typical readers *M* = 9.9, *SD* = 7.37; *t*(32.806) = 4.271, *p* < 0.001, *d* = 1.182). These ADHD scores were also correlated with ARHQ, reading speed, and reading accuracy (all absolute *r*s > 0.254, all *p*s < 0.05 except for ADHD-I and reading accuracy, *r* = -0.153, *p* = 0.244). Those who reported more symptoms of ADHD tended to have a greater history of reading problems and read slower and less accurately.

In the analyses to follow, we estimate to which degree ADHD measures (current ADHD symptoms, childhood ADHD symptoms, ADHD diagnosis) can account for other patterns in our data. For comparison of data with and without the exclusion of participants with a previous ADHD diagnosis, see Supplementary Information.

### Other disorders

No participants reported a previous diagnosis of autism spectrum disorders or language disorders other than dyslexia. One typical reader reported poor hearing, and two dyslexic readers reported being dyscalculic. These participants were included in the sample but excluding them would have minimal impact on our analyses.

### Face matching

#### Overall group differences and correlations

**Figure 2:**
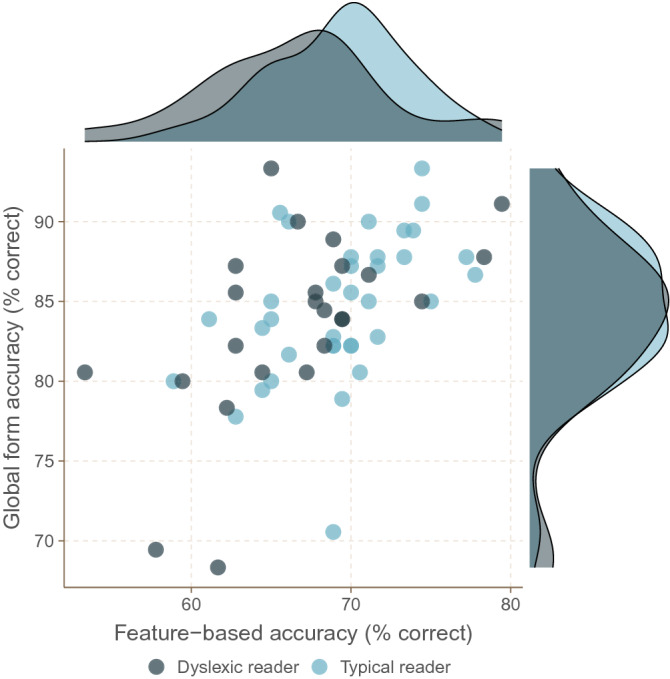
Performance of dyslexic (black) and typical (blue) readers on feature-based and global form face matching. Each dot corresponds to one participant. Marginal plots show density estimates for the two groups.

As seen in Fig. 2, dyslexic readers as a group were less accurate than typical readers on feature-based face matching but not global form face matching (feature-based: dyslexic readers *M* = 66.6%, *SD* = 5.98; typical readers *M* = 69.3%, *SD* = 4.33; *t*(58) = 2.029, *p* = 0.047, *d* = 0.517; global form: dyslexic readers *M* = 83.7%, *SD* = 5.87; typical readers *M* = 84.6%, *SD* = 4.55; t(58) = 0.733, *p* = 0.466, *d* = 0.188). The null result for global form but not in feature-based face matching could not be explained by a difference in task reliability, as the global form face matching task was slightly more reliable (*α* = 0.778) than the feature-based face matching task (*α* = 0.664) while it is generally easier to detect a group difference with a more reliable measure.

Feature-based matching was overall harder than matching based on global form. Our previous data^41^ suggest that difficulties with face matching consistently predict dyslexia over and above two other visual tasks of equal or greater difficulty, which speaks against the possibility that feature-based face processing problems were found simply because the task was hard. However, we sought to rule out this possibility by further analyzing the feature-based trials, as some were consistently harder than others. If dyslexic readers are simply bad at difficult visual tasks, then the typical reader advantage would be expected to be greater for hard feature-based trials as compared to easy feature-based trials, but this was not the case. Contrary to this possibility, trial difficulty in the feature-based face matching task (per trial mean accuracy across groups with both groups given equal weight) was uncorrelated with the typical reader advantage (per trial typical reader mean accuracy minus dyslexic reader mean accuracy for that same trial), *r*(178) = − 0.004, *p* = 0.959. The group effect was therefore unlikely due to the feature-based task simply being more difficult than the global form face matching task.

We found an expected group effect of decent size (a medium effect size according to traditional criteria) for feature-based face matching accuracy. Nonetheless, zero-order correlations between feature-based face matching accuracy and our three primary reading measures did not reach significance (all absolute *r*s < 0.168, all *p*s > 0.201). We suspected that this association was being masked by other variables. More specifically, we reasoned that accuracy for feature-based face matching measured a combination of task-specific effects—of primary interest—as well as other variables not of primary interest. This is evidenced by the positive correlation between feature-based and global form face matching accuracy (Fig. 2). Such shared variance can be due to several things, among them: non-verbal IQ, attention and vigilance, non-specific visual mechanisms, face-specific visual mechanisms, emphasis on speed vs. emphasis on accuracy etc. Similarly, many things can be associated with reading problems, including the well-known connection between ADHD symptoms and reading deficits as is indeed seen in the current study. It is therefore important to isolate components of feature-based face matching and reading problems not explained by these potential common factors. As detailed below, the connection between feature-based processing difficulties and reading problems is likely quite specific as it becomes clearer after partialling out non-specific factors (see also Supplementary Information: ADHD Measures).

#### Specific effects: regression models

To focus on task-specific effects, we performed a hierarchical (sequential) logistic regression with group membership (0: typical reader; 1: dyslexic reader) as the dependent variable and three hierarchical linear regression models with scores on the Adult Reading History Questionnaire (ARHQ), reading speed, and reading accuracy as dependent variables. Given the well-known association between ADHD and reading problems (see Results: ADHD Measures) and given that inattention could plausibly affect face matching performance, we entered ADHD-I and ADHD-II scores as well as previous ADHD diagnosis (0: not diagnosed; 1: diagnosed) at the first stage of each model. Global form face matching accuracy (% correct) and face matching response times were entered on the second stage. Face matching response times were calculated by taking the mean z-score of feature-based and global form face matching response times as the two were highly correlated (*r* = 0.956, *p* < 0.001). Feature-based face matching accuracy (% correct) was entered at the third and last stage.

The logistic regression model at stage 1 was significant, $\chi^{2}$(3) = 23.739, *p* < 0.001, *R*^2^_Nagelkerke_ = 0.442. At stage 1 of the linear regression models, ADHD measures also explained a significant amount of the variance in ARHQ scores (*F*(3,56) = 9.301, *p* < 0.001, *R*^2^ = 0.333, *R*^2^_*adjusted*_ = 0.297) and reading accuracy (*F*(3,56) = 3.268, *p* = 0.028, *R*^2^ = 0.149, *R*^2^_*adjusted*_ = 0.103), but not reading speed (*F*(3,56) = 2.383, *p* = 0.079, *R*^2^ = 0.113, *R*^2^_*adjusted*_ = 0.066). As expected, ADHD measures were therefore highly predictive of dyslexia and of reading problems in general.

The addition of global form face matching accuracy and face matching response times at stage 2 did not improve any models (model change for group membership: $\chi^{2}$(2) = 0.786, *p* = 0.675, *R*^2^_Nagelkerke_ change = 0.012; for ARHQ: *F*(2,54) = 0.268, *p* = 0.766, *R*^2^ change = 0.007; for reading speed: *F*(2,54) = 0.351, *p* = 0.706, *R*^2^ change = 0.011; for reading accuracy: *F*(2,54) = 1.316, *p* = 0.277, *R*^2^ change = 0.040).

Adding the feature-based face matching accuracy at stage 3 significantly improved all models (model change for group membership: $\chi^{2}$(1) = 7.559, *p* = 0.006, *R*^2^_Nagelkerke_ change = 0.106; for ARHQ: *F*(1,53) = 9.114, *p* = 0.004, *R*^2^ change = 0.097; for reading speed: *F*(1,53) = 6.002, *p* = 0.018, *R*^2^ change = 0.089; for reading accuracy: *F*(1,53) = 5.319, *p* = 0.025, *R*^2^ change = 0.074). When all other variables were held constant, lower feature-based face matching accuracy was associated with an increased likelihood of being dyslexic, a greater history of reading problems, and slower and less accurate reading. Poorer task-specific performance for feature-based face matching was therefore associated with poorer reading-specific measures. The final stage 3 models are summarized in Table 1 (see also Supplementary Material S4. Regression Models Accounting for Visual Search).

**Table 1:** Summary of regression models with group membership (logistic regression, $\chi^{2}$(6) = 32.085, *p* < 0.001, *R*^2^_Nagelkerke_ = 0.560), ARHQ (linear regression, *F*(6,53) = 6.831, *p* < 0.001, *R*^2^ = 0.436, *R*^2^_*adjusted*_ = 0.372), reading speed (linear regression, *F*(6,53) = 2.400, *p* = 0.040, *R*^2^ = 0.462, *R*^2^_*adjusted*_ = 0.214) and reading accuracy (linear regression, *F*(6,53) = 3.145, *p* = 0.010, *R*^2^ = 0.263, *R*^2^_*adjusted*_ = 0.179) as dependent variables, and measures of ADHD and face processing as independent variables. Unstandardized regression coefficients (*b)* and *p*-values are bold for significant independent predictors.

	Group		ARHQ		Reading speed		Reading accuracy	
	*b*	*p*	*b*	*p*	*b*	*p*	*b*	*p*
Constant	11.503	0.157	**0.744**	**0.008**	− 10.859	0.338	**0.619**	** < 0.001**
ADHD-I	0.090	0.210	0.005	0.212	0.010	0.983	0.002	0.166
ADHD-II	**0.118**	**0.019**	**0.005**	**0.046**	− 0.416	0.143	− **0.003**	**0.002**
ADHD diagnosis	21.472	0.999	**0.297**	**0.009**	− 23.275	0.085	0.012	0.754
Face matching RT	0.585	0.234	0.042	0.094	− 5.795	0.060	− **0.021**	**0.018**
Global form accuracy	0.058	0.525	0.007	0.133	− 0.134	0.804	0.001	0.623
Feature-based accuracy	− **0.291**	**0.019**	− **0.015**	**0.004**	**1.504**	**0.018**	**0.004**	**0.025**

#### Laterality effects

**Figure 3:**
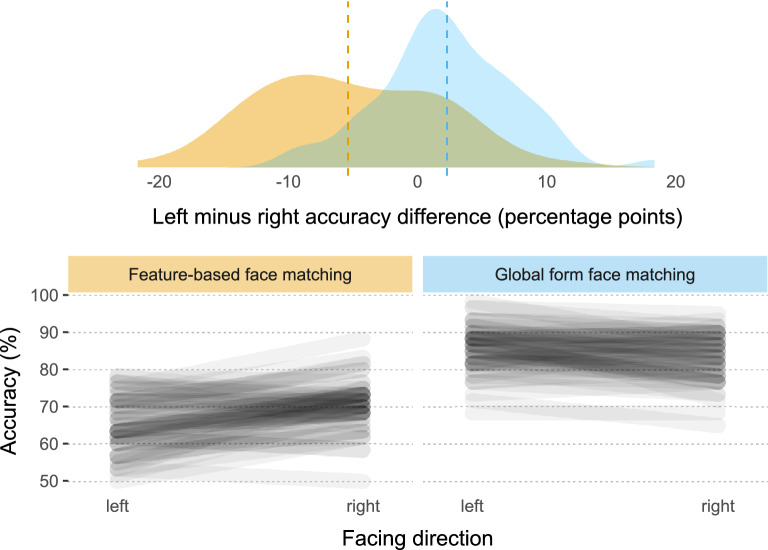
Performance by orientation of faces (facing direction). Accuracy for feature-based face matching was poorest for leftward-facing stimuli and greatest for rightward-facing stimuli. The opposite laterality pattern was seen for global form face matching. **Upper panel:** Density estimates for laterality effects (percent correct for left-facing stimuli minus percent correct for right-facing stimuli) in feature-based face matching (yellow) and global form face matching (blue). Mean laterality effects are shown as vertical dashed lines. **Lower panels:** Individual participants’ scores for left-facing and right-facing stimuli in feature-based face matching (left panel) and global form face matching (right panel) are shown as connected lines.

Exploratory analysis (hence no *p* values, as they lose their meaning when not doing confirmatory hypothesis testing^84,85^; for a partial rebuttal, see^86^) revealed opposite laterality effects (Fig. 3) for feature-based and global face processing (see also Supplementary Information: Laterality Effects) based on facing direction to the best of our knowledge previously undocumented in the literature even for typical readers. As described in the Methods section, the three faces shown on each trial were all oriented in the same direction, which could be 30° leftward, forward, or 30° rightward. In feature-based face matching, average performance was noticeably better on right-facing (*M* = 70.5%) than left-facing (*M* = 65.1%) trials (*d* = 0.778). The opposite was true for global form face matching, where people tended to perform better on left-facing (*M* = 85.6%) as compared to right-facing (*M* = 83.3%) trials (*d* = 0.431). Both laterality effects were consistently seen as witnessed by their moderate-to-large effect sizes. The effect size estimate for the difference in these laterality effects for the two types of face matching trials was even larger (*d* = 0.968). There was however not a strong correlation between the two effects (left minus right accuracy difference for feature-based vs. for global form face matching, *r* = 0.180) which could indicate that they are independent of each other.

**Figure 4:**
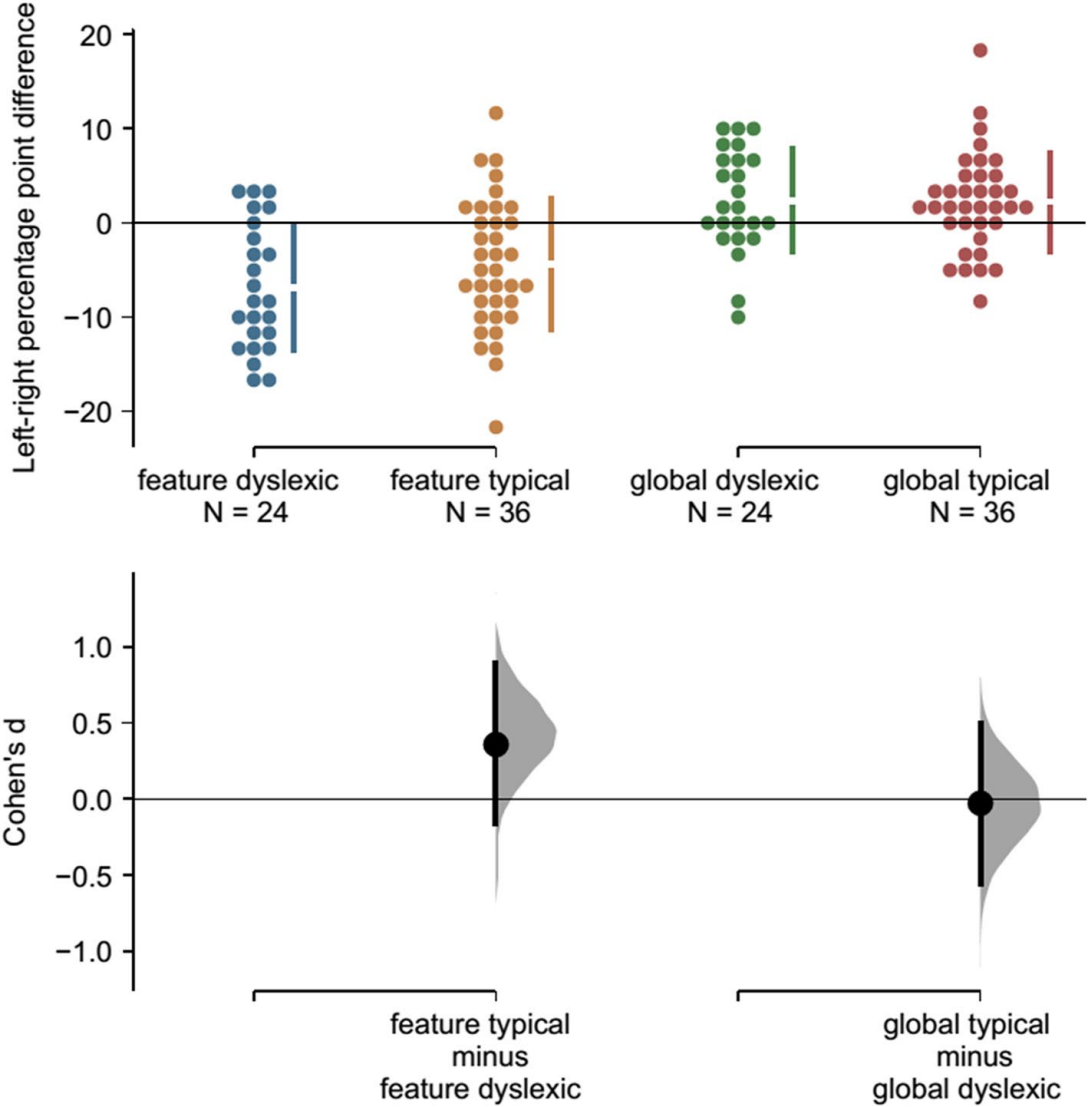
Cumming plot depicting laterality effects of dyslexic (blue and green dots) and typical readers (yellow and red dots). **Upper panel:** Each dot corresponds to one person’s laterality effect (accuracy for left-facing stimuli minus accuracy for right-facing stimuli); means and standard deviations are plotted as gapped lines. **Lower panel:** Effect sizes (Cohen’s d) are depicted as dots. Filled curves depict the resampled distribution of the group differences, given the observed data, and error bars represent 95% confidence intervals (bootstrapped). Image is based on code developed by Ho, Tumkaya, Aryal, Choi, and Claridge-Chang^87^.

Laterality effects for global form face matching were seen in both groups to a similar degree (dyslexic readers *d* = 0.430; typical readers *d* = 0.425) while laterality effects for feature-based face matching were numerically somewhat larger for dyslexic readers compared to typical readers (dyslexic readers *d* = 1.030; typical readers *d* = 0.628; Fig. 4). It should be noted that while feature-based face matching group differences in the sample were moderate for leftward-oriented faces (*d* = 0.443) and small for rightward-oriented faces (*d* = 0.119), they were most prominent for frontal faces (*d* = 0.639), and adding the laterality effect as an additional covariate in the regression models summarized in Table 1 had essentially no effect on the reported specific association with overall feature-based face matching accuracy. Further exploratory analysis hints at the possibility that feature-based laterality effects, unlike overall weaknesses in feature-based visual processing, might not be specifically related to dyslexia but could instead be more associated with individual differences in reading performance in typically developing people, which could ultimately reflect differences in their reading experience, see Supplementary Information: S2 Laterality Effects and supplementary Fig. S3. Further studies are needed to test by confirmatory data analysis whether these opposite laterality effects for feature-based and global form face processing, group differences in feature-based laterality, and associations with reading are real.

## Discussion

The current study indicates that dyslexic readers tend to be worse at feature- or part-based processing of faces compared to typical readers, while no group differences were found in global or configural processing of faces. Establishing such a specific feature-based processing deficit was the main reason for conducting this study as it is of theoretical importance for theories on the domain-specificity vs. domain-generality of visual word and face processing as well as for our high-level visual dysfunction hypothesis of developmental dyslexia.

The study shines a light on the codependence versus independence of visual word and face processing, and more generally on domain-specificity vs. domain-generality within the visual system. Traditionally, words and faces are thought to be independently processed, perhaps even in independent cortical regions of the two hemispheres, words in the left hemisphere and faces in the right (see e.g.^17,88^). According to the many-to-many hypothesis^7^, no single brain region is however responsible for the visual recognition of objects such as faces or words. Instead, overlapping, distributed, bilateral brain circuits mediate the recognition of both object classes. Our results support the many-to-many view that faces and words share common neural resources within the ventral visual stream.

However, the many-to-many view should be further constrained by the type of processing involved. Here we show that processing the global form of faces apparently shares minimal—if any—resources with visual word processing, while word and face perception are associated when the latter requires the processing of fine-grained visual features of a face (for related work on brain damaged patients, see e.g.^89,90^).

The current results are at odds with the prediction of Dehaene et al.^11^ of an inverse relationship between face and word recognition, as reading skills either have no relationship with face perception abilities (global form face matching) or a positive relationship with face perception abilities (feature-based face matching). More generally, they go against the destructive-competition version of the neuronal recycling hypothesis^11,27^ which suggests that words encroach on cortical space and computational resources that otherwise would have been dedicated to objects such as faces to the detriment of their processing; for similar conclusions based on research on illiterates, see van Paridon et al.^91^.

The fact that reading problems are associated with a specific feature-based face processing deficit can be compared with acquired and developmental prosopagnosia. The face specificity of prosopagnosia, like the word specificity of acquired and developmental reading problems, has long been debated. A particular disruption of global/configural/holistic processing has however been reported in prosopagnosia (although the specificity of this effect might be better established for acquired prosopagnosia^92–99^). The current results are consistent with the intriguing possibility that prosopagnosia and our hypothesized high-level visual dysfunction subtype of developmental dyslexia are essentially mirror versions of each other (see^7^). This needs further validation.

Our results on laterality effects were exploratory and need to be interpreted with caution. As it has long been debated whether faces and words are primarily processed in opposite hemispheres, these laterality effects nonetheless warrant further discussion (see also Supplementary Information: S2 Laterality Effects). Furubacke et al.^13^ have already called for a modification of the many-to-many hypothesis so as to take laterality of function into account. They report that visual face and word processing share resources only when tasks rely on the same hemisphere—focusing on face identity thus shares some resources with focusing on handwriting, as both rely on right hemisphere processing, and focusing on word identity shares resources with focusing on facial speech sounds/lip reading, as they tap into left hemisphere processing (for more information on left hemisphere processing of lip reading and audio-visual integration of speech, see e.g.^100–102^). While a large body of research suggests that the right hemisphere is highly important for identifying faces and the left hemisphere for identifying words, face-responsive and word-responsive visual regions are nonetheless found bilaterally^2^ and unilateral lesions can lead to simultaneous face and word recognition deficits^19^. In accordance with some other literature (see Supplementary Information: S2 Laterality Effects for further discussion), the current results suggest that both hemispheres support the discrimination of faces but to a different degree depending on the type of processing.

Global form face processing laterality effects were consistent with a right hemisphere lateralization, and we found no evidence for overall group differences (dyslexic vs. typical readers) in lateralization for this task. This can be contrasted with ideas of the joint development of hemispheric lateralization for words and faces, where the general left visual field (right hemisphere) superiority for faces is reportedly associated with greater reading abilities and has been suggested to be driven by left hemisphere word lateralization^103^ (see also^7,11,29,104,105^). This also seems somewhat at odds with previous work where a left visual field (right hemisphere) advantage for faces was reported for typical readers while no apparent face lateralization was found for dyslexic readers^14^ or where no consistent face lateralization was found in either group^48^. Further exploratory analysis however does hint at a possible difference between typical and dyslexic readers in the relationship between reading and the lateralization of global form face processing, see Supplementary Information: L2 Laterality Effects and Supplementary Fig. S3. This needs to be independently replicated.

For feature-based face matching, both groups showed laterality effects consistent with left hemisphere lateralization. This effect was particularly strong in the dyslexic group (d > 1). The replicability of this exploratory analysis should be independently verified, but it is possible that weaknesses in reading are associated with greater left hemisphere lateralization of feature-based face processing. This could be consistent with a weaker left-side bias for Chinese character recognition of dyslexic readers in Hong Kong, likely indicating lessened right hemisphere lateralization/greater left hemisphere lateralization^106^. The authors suggest that dyslexic readers may not form appropriate part-based representations in the right hemisphere.

Further exploratory analysis provides hints that laterality of feature-based face processing might not be specifically related to dyslexia but instead to reading performance and experience (see Supplementary Material S2. Laterality Effects). There was a positive correlation between reading speed and feature-based laterality effects specifically in the typical reader group (Supplementary Fig. S3), which is consistent with faster readers being less left hemisphere lateralized/more right hemisphere lateralized in feature-based face matching. This was mostly driven by associations with the reading of unfamiliar material, i.e., pseudowords and uncommon real words. If this is a true effect—which needs to be independently verified—this could be in accordance with the possibility that in typical reading development, very common words are read comparatively more holistically/globally (for evidence for holistic word processing in expert readers, see e.g.^69,70,107,108^) while unfamiliar material is read more piecemeal/featurally, and that this feature-based processing of written material specifically competes with feature-based face processing, originally left-lateralized but becomes less so due to this competition. According to the neuronal recycling hypothesis, cultural inventions such as reading lead to the invasion of evolutionarily older cortical precursor maps with anatomical and connectional constraints that fit the new skill^27^. If these associations between reading and laterality of feature-based processing are replicable and real, this could be in accordance with reading experience specifically leading to competition for cortical “real estate” between feature-based face and word processing in high-level ventral visual regions of the left hemisphere^11,27^, consistent with the likely important role of feature-based processing of words.

Our ideas have ever since the beginning been guided by the possibility that high-level visual problems associated with dyslexia might be feature-based and left-lateralized (for further discussion, see^43^). The current results fit well with our suggestion of the former but not the latter. The dyslexic group had specific weaknesses in feature-based processing of faces. However, overall group differences in feature-based face processing accuracy were seemingly independent of any differences in laterality effects and were, if anything, smallest for rightward-oriented faces (assumed left hemisphere processing). These results fit better with reports on people with a posterior cerebral artery stroke, where patients with word recognition difficulties could also have problems in face recognition independently of the affected hemisphere^109^ (see also^19^). There are also reports of abnormal processing of faces in the bilateral ventral stream of impaired readers^110^, primarily in the right hemisphere^105^, and in the left hemisphere^39^. A possible bilateral processing deficit does not necessarily go against the idea of a feature-based processing deficit as the right hemisphere has been suggested to flexibly switch between holistic and part-based representations depending on the type of information being used^111^. For example, expert readers of Chinese characters process them less holistically than novices—consistent with the importance of featural information in Chinese character recognition—yet show hints of increased recruitment of right hemisphere regions for these characters^112,113^, while experts in recognizing Greebles (computer-generated novel objects) show increased holistic processing of these objects as well as increased recruitment of right hemisphere regions (in the fusiform face area)^114^. It is possible that the right hemisphere becomes sensitive to whatever information is most diagnostic for a particular object class. Regardless of possible hemispheric differences, our results are consistent with unusual or faulty high-level visual mechanisms in dyslexia, which we suggest here are specifically feature-based and not global or configural.

While dyslexia was originally considered a visual deficit^115–118^, the focus of dyslexia research moved from perceptual-based theories to language-based theories, particularly to the processing of phonological information (e.g.^119^; for an overview, see^33^). The evidence for phonological problems in dyslexia is strong, and it is not our intent to suggest that a visual theory of reading problems should replace the phonological view or other evidence-based views. However, several factors could contribute to reading problems, and interest in the contribution of visual factors has recently resurfaced. Our high-level visual dysfunction hypothesis is a new idea on the causes of reading problems and its empirical testing is thus greatly needed.

The current study suggests that reading problems are independent of the processing of global form but associated with weaknesses in feature-based processing, generally believed to play a much larger role in reading. This is consistent with a high-level visual dysfunction subtype of developmental dyslexia characterized by weaknesses in feature-based processing. It should nonetheless be explicitly stated that we found an association and not direct evidence for a causal role in developmental dyslexia. Finding such a group effect does not indicate that all dyslexic readers have “ventral visual stream problems” nor does it indicate that those who potentially do would have crippling face processing deficits in everyday life. It also does not indicate that high-level visual problems go hand in hand with all reading problems. Reading is a complex skill which can be broken down into several subskills, only some of which might be expected to have anything to do with visual cognition. Our reading measures were specifically focused on visual word decoding, and not on reading comprehension, as visual perception mechanisms are more likely to partake in the former than in the latter. This is also consistent with the fact that people with developmental dyslexia have difficulties with accurately and fluently recognizing and decoding words, while people with specific reading comprehension deficits have poor reading comprehension despite adequate word recognition and decoding abilities, and only the former group but not the latter shows abnormal functioning in high-level regions of the ventral visual stream^120^.

There are some reasons to believe that problems with feature-based face processing might be underestimated in the current study. First, the sample included only current or former university students. Dyslexic university students might have less profound difficulties in reading compared to those who do not pursue a university degree, and have more experience with written words, making them distinct from dyslexic readers in general. Our previous research indicates that face-processing deficits might be most pronounced for less educated dyslexic readers^42^. Secondly, while ARHQ is an excellent screening tool for dyslexia, it is always possible that some people who screened positive for dyslexia in this study would not meet formal diagnostic criteria; such group misplacements could attenuate group differences. Third, the reliability for feature-based face matching was questionable, so estimates of individual differences in feature-based processing were noisy which would be expected to diminish measured effect sizes. Finally, stimuli were computer-generated images of faces which are arguably less detailed than real faces, which could diminish the feature-based processing of these faces as compared to real faces or have other unforeseen effects such as making it harder to apply previous visual experience with real faces (then again, the journal’s quality check flagged the face images as identifying participants and wanted them removed, so they seem real enough). It would be good to replicate the current study in a more diverse sample and with other stimuli.

A local-before-global precedence has been reported for dyslexic readers in the Navon task^121^. The authors suggest that a decreased weighting or reliance on global information in dyslexic readers could be due to a dorsal stream deficit, as the dorsal stream is specialized for low spatial frequency/global processing. Similarly, Schmitt et al.^122^ report that dyslexic children, unlike controls, show no global-to-local interference in the Navon task which the authors interpret as an overreliance on analytic processing as opposed to holistic or global processing. Conversely, compared to typical readers, dyslexic readers showed signs of stronger holistic processing of English words^71^ and Chinese characters^106,123^, and illiterates process both faces and houses more holistically compared to controls^124^. It is unclear whether these results are inconsistent with our findings of intact global form face processing in dyslexic readers. Inducing a global vs. local bias with Navon stimuli has both been reported to affect^125,126^ and not affect^127^ face processing. The visual processing of alphabetical scripts, such as Icelandic, and logographic scripts, such as the Chinese writing system, could be qualitatively different. Chinese character recognition also differs from face recognition as it requires extensive writing practice; writing experience could decrease holistic processing of such characters^106,123,128^ in a similar way that face drawing experience appears to decrease the holistic processing of faces^129^. While increased holistic processing of English words in English-speaking dyslexic readers is a bit harder to reconcile with our results, the same authors reported similar holistic processing of faces for typical and dyslexic readers^71^, and impaired holistic word processing in dyslexia has also been reported^72^. Brady et al.^71^ suggest that typical readers may switch more easily between holistic and analytic processing as required (see also^130^). Finally, the finding that adult dyslexic readers, unlike illiterate people^124^, show no signs of increased holistic processing of faces^14,43,71^, might be due to their nonetheless extensive lifelong experience with reading.

Aaron^46^ compared subgroups of dyslexic children classified either as dysphonetic or dyseidetic based on their spelling errors (see also^131^) and found that the latter group identified significantly fewer faces than the former. According to Boder^131^, dysphonetics read words globally as instantaneous visual Gestalts rather than analytically as they are unable to sound out and blend the component letters and syllables of a word. On the other hand, dyseidetics, which Aaron^46^ refers to as holistic-simultaneous deficient, supposedly have poor memory for visual Gestalts, so they read analytically by sounding out the letters. While this fits broadly with some sort of “phonological” vs. “visual” subtypes of dyslexia, why would dyslexic readers with a supposedly holistic visual deficit have more problems with face recognition when the current results indicate that holistic or global processing of faces is apparently unrelated to reading deficits? This might stem from terminological confusion—and here we do not exclude ourselves—as holistic processing is too loosely defined^66^. The dyseidetic dyslexic children in Aaron^46^ were simply grouped as such as they made phonologically logical spelling errors, such as writing “tebl” instead of “table”. While this could be indicative of sufficient phonological processing ability, it says little about whether their spelling errors and face recognition difficulties were due to problems with holistic processing, feature-based processing, or something else entirely.

Feature-based processing, often used synonymously with analytical processing (e.g.^132^), might similarly have multiple meanings. Analytical processing can be defined as processing an object in terms of its individual components^132^, focusing on a single feature^133^, processing a dimension of an object without being influenced by other dimensions or features^134^, the whole simply being the sum of its parts^135^, and explicit structural descriptions based on an object’s parts and their relations^136^, to name a few examples. While these surely have a common theme, they do not necessarily completely overlap; for example, focusing on a single feature seems to imply sequential processing of features, while processing individual components might or might not be done in parallel, and a structural description might by some be called configural instead of featural. The main difference between holistic and feature-based processing might even be that the former simply makes use of larger visual features^137^.

We operationally defined feature-based and global form processing based on particular stimulus manipulations. This does not ensure that these actually lead to qualitative differences in visual processing. We are nonetheless convinced that feature-based and global form face matching trials partially tap into different processing mechanisms. First of all, the two stimulus manipulations of this exact stimulus set have been previously shown to be differentially impacted by face inversion^78^, commonly thought to affect configural or holistic processing more than feature-based processing (but see^138^). Secondly, the opposite laterality effects for feature-based and global form face matching seen in the current study makes the case for different processing mechanisms even stronger. While laterality effects in face perception are well-known (for an overview, see^56^), the opposite laterality effects of feature-based and global form face processing based on facing direction seen in this study have to the best of our knowledge not been previously documented even in typical readers, yet are in alignment with what is already known about hemispheric differences where feature-based face processing is thought to rely more on the left hemisphere compared to global form face processing (for further discussion, see Supplementary Information: S2 Laterality Effects). The two subtasks are most likely tapping into two partially separable mechanisms that rely to a different extent on the two cerebral hemispheres.

We nonetheless explicitly want to say that it is impossible to know exactly what constitutes these qualitative differences; we simply do not know whether facial features in feature-based face matching were processed serially or in parallel, independently or whether they interacted with one another etc. It also remains to be seen whether the supposed feature-based face matching problems of dyslexic readers are more accurately described as reduced sensitivity to higher spatial frequency information in faces, as matching faces based on configural information is more efficient for low spatial frequency faces, while matching faces based on differences in their features is largely depends on high spatial frequencies^139,140^ (but see^141^). This would fit well with results from alexic patients with lesions involving the left fusiform gyrus who were impaired at matching faces for identity across viewpoints, especially when faces were reduced to line contours (primarily high spatial frequency information)—while a prosopagnosic patient with a corresponding right hemisphere lesion did well with line-contour images^100^. Even if this was the case, we would not expect this to be due to low-level visual problems as we have previously shown that difficulties with face matching predicted dyslexia over and above matching noise patterns that shared such low-level visual properties with faces^41^. Exactly pinning down the high-level visual mechanisms deficient in some dyslexic readers is a project worthy of further study (for follow-up work, see^130^).

People have long debated the nature of visual object recognition abilities. Are there specific modules that only deal with particular object categories such as faces and words, or are there instead subsystems needed to recognize objects by extensive part decomposition (e.g. words) vs. little or no part decomposition (e.g. faces;^26^)? Our results are consistent with this latter interpretation, as visual word and face processing are both associated and dissociated—but this depends on what kind of visual mechanisms are task-relevant. The current results furthermore suggest that reading problems have more than one underlying factor, and that visual as well as non-visual mechanisms could play a role. We suggest that a subtype of developmental dyslexia is characterized by a high-level visual dysfunction, in particular by weaknesses in feature-based visual processing.

## Supplementary Information

Supplementary Information.

## Data Availability

Data can be made available to other researchers upon request provided that the National Bioethics Committee of Iceland grants them permission for such access and provided that such access adheres to all Icelandic laws regarding data privacy and protection.
