# Supplementary material for: Faces and words are both associated and dissociated as evidenced by visual problems in dyslexia
Source: Sci Rep. 2021 Nov 26;11:23000. doi: 10.1038/s41598-021-02440-7 (PMC8626489; doi:10.1038/s41598-021-02440-7)
Supplement: Supplementary file 1 — Supplementary Information. [file 41598_2021_2440_MOESM1_ESM.pdf]

Supplementary Information

S1. Reading Speed and Accuracy by Group

Reading speed and reading accuracy can be seen separated by group membership (dyslexic vs. typical readers) in figures s1 and s2, respectively. As we separate the lists here instead of combining them, reading speed and accuracy values for a missing list were dropped instead of imputed from the reading speed and accuracy of the two other lists, unlike in the main manuscript.

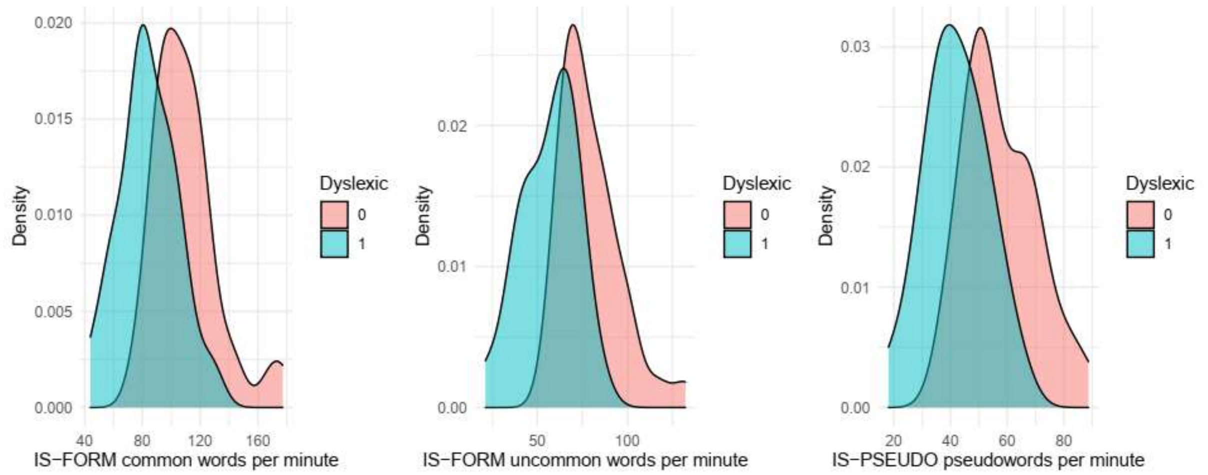

Figure s1. Reading speed for the three reading lists separated by group (cyan: dyslexic readers; pink: typical readers). Please note scale differences.

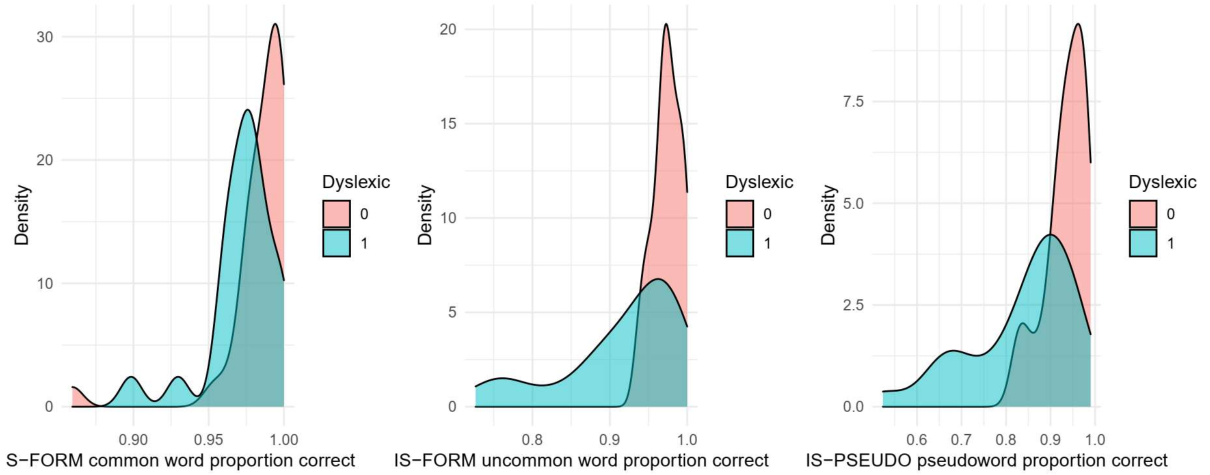

Figure s2. Reading accuracy for the three reading lists separated by group (cyan: dyslexic readers; pink: typical readers). Please note scale differences.

S2. Laterality Effects

All other things equal, a face will be processed more efficiently if it appears in the left visual field (projecting to the right hemisphere) compared to the right visual field (projecting to the left hemisphere; for discussion on contralateral biases in high-level visual cortex, see e.g. Hemond, Kanwisher, & De Beeck, 2007; Kay, Weiner, & Grill-Spector, 2015; Pitcher et al., 2019). People are also generally likely to report that a chimeric face made of two left halves, rather than right halves, of an original face image resembles the original, presumably again because the left half of the original face image tends to fall within the left visual field (for a review on lateralization of face processing, see Sigurdardottir & Jozranjbar, 2019). However, such effects appear to be dependent on the type of processing involved. A right visual field (left hemisphere) advantage in a same-different task for faces has been reported when faces can only be distinguished based on a single feature (e.g. different eyes, same nose and mouth; Fairweather et al., 1982; Hillger & Koenig, 1991) or possibly one or two features (Sergent, 1982), and has been attributed to the requirements for parsing and analyzing the local elements of a face (Hillger & Koenig, 1991).

In the current study, faces were always shown simultaneously in three locations (left, center, right) and their orientation could be toward the left, right, or straight ahead. Average performance in feature-based face matching was noticeably better on right-facing than left-facing trials. The opposite was true for global form face matching, where people tended to perform better on left-facing as compared to right-facing trials. There are at least two possible drivers of these effects (assuming that they replicate). It is possible that the main diagnostic “face” part of a left-facing central sample stimulus tends to fall within the left visual field, and that this in turn triggers preferential right hemisphere processing, while the opposite could be true for

a right-facing sample. Another possibility is that faces oriented to the left engage attentional deployment toward the left visual field, while a rightward orientation of faces directs attention to the right visual field, as gaze evokes fast reflexive attentional shifts (Driver et al., 1999; Friesen & Kingstone, 1998; see also Sigurdardottir, Michalak, & Sheinberg, 2014). Lateralized attentional deployment could suffice to engage partially lateralized feature-based vs. configural processing mechanisms as neurons high up in the ventral visual stream mainly process objects at the center of attention (Brooks, Sigurdardottir, & Sheinberg, 2014).

Laterality effects in face matching were found through exploratory data analysis and should be interpreted with caution, including the following exploratory analyses.

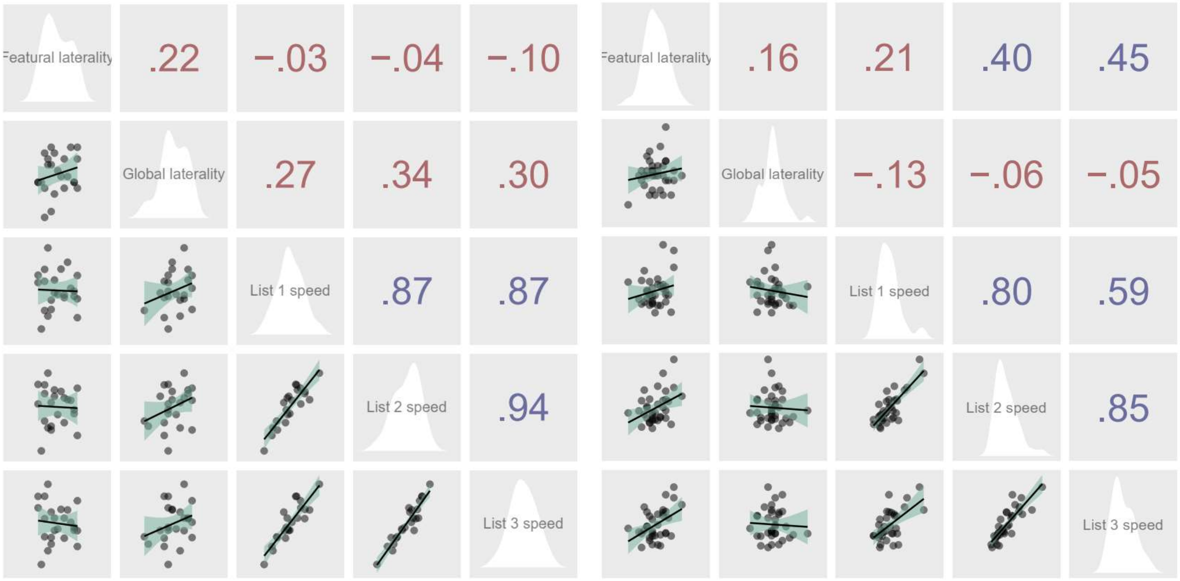

Figure s3. Correlations for **dyslexic readers (left panel)** and **typical readers (right panel)** between reading speed (list 1: IS-FORM common word forms; list 2: IS-FORM uncommon word forms; list 3: IS-PSEUDO pseudowords) and laterality effects (percent correct for left-facing stimuli minus percent correct for right-facing stimuli) for feature-based face matching (featural laterality) and global form face matching (global laterality). Blue correlation coefficients are significant, and red are non-significant according to traditional criteria, but readers should keep in mind that this analysis is exploratory, and that the dyslexic group has fewer participants.

As mentioned in the main manuscript, deficient feature-based processing in dyslexic readers appeared to be independent from any possible group differences in feature-based face processing laterality effects. Feature-based laterality effect might not be specifically related to dyslexia but instead to reading performance and experience. To explore this possibility, we correlated the feature-based laterality effects with reading speed and accuracy of the three reading lists (IS-FORM common word forms, IS-FORM uncommon word forms, IS-PSEUDO pseudowords) separately for the two groups of typical and dyslexic readers. Laterality indices below zero are consistent with left hemisphere lateralization and those above zero are consistent with right hemisphere lateralization.

There was no detectable association between reading accuracy and feature-based laterality effects in either group (all absolute rs < 0.22). This could be due to truncated range of reading accuracy, particularly in the typical reader group as most typical readers read quite accurately while there was more variability in reading accuracy in the dyslexic group, see supplementary figure s2. In the dyslexic group, correlations between reading speed of the three reading lists and feature-based laterality effects were small and negative (supplementary figure s3, left panel). However, there was a positive correlation between reading speed and feature-based laterality effects in the typical reader group (supplementary figure s3, right panel). We discuss this association in the Discussion chapter of the main manuscript.

It is also interesting to look at possible group differences in correlations with global form laterality effects. While correlations with global laterality indices are small and negative in the typical reader group, they are somewhat larger and positive in the dyslexic reader group. So, unlike that for typical readers, faster reading of dyslexic readers is possibly associated with increased right hemisphere lateralization of global

form face processing. This is very speculative, but it could be in accordance with the possibility that in order for dyslexic readers to gain more reading competence, they need to compensate for a feature-based processing deficit by implementing reading strategies that tap into other types of processing such as global or holistic processing. We note that this is reminiscent of the results of Brady et al. (2020), who report that greater holistic processing predicts better reading of dyslexic readers but worse reading for typical readers.

We finally want to mention that the reading speed of all three lists is highly correlated in the dyslexic group while individual differences in the typical reader group seem to separate into the reading of familiar and unfamiliar material, as evidenced by the lower correlation between reading speed for common word forms and pseudowords (supplementary figure s3). This fits with the possibility that while typical readers rely on different strategies for the reading of familiar and unfamiliar material, dyslexic reader might rely on a similar mechanism in both cases. Skilled reading has been suggested to involve flexibly applying several reading subskills, such as logographic, alphabetic, and orthographic, depending on the requirements of the reading task (Lachmann, 2018). The inability to do so may impair reading.

S3. ADHD Measures

Table s1 shows the results for simple group comparisons of performance in face matching with and without participants who reported a previous ADHD diagnosis. Excluding these participants slightly increased the group difference for feature-based face matching, but otherwise had minimal effect.

Table s1. Simple group comparisons of visual measures. The table shows p-values from independent samples t-tests (with adjusted degrees of freedom in cases where Levene's test was significant) and Cohen's d effect sizes. Significant group differences are underlined in bold.

	All participants (N = 60)		Without ADHD diagnosis (N = 58)	
	p	*d*	*p*	*d*
Face matching RT	0.491	0.184	0.336	0.266
Global form accuracy	0.466	0.188	0.400	0.221
Feature-based accuracy	**0.047**	**0.517**	**0.013**	**0.668**

The ADHD-I and ADHD-II questionnaires were not significantly correlated with performance in face matching (table s2). ADHD-I and ADHD-II scores were on the other hand significantly correlated with several reading measures (table s3). There was, in other words, a significant proportion of the variance in reading problems that could be statistically accounted for by symptoms of ADHD. This highlights that it might not be sufficient, as done in some studies, to simply exclude participants with a previous ADHD diagnosis, as ADHD symptoms are still associated with reading problems even if participants with such a diagnosis are excluded, so a modulatory role of ADHD symptoms in such studies cannot be ruled out.

Table s2. Correlation (Pearson's r) between several visual measures and Behavioral Evaluation Questionnaire for Adults I and II, intended to assess symptoms of ADHD. No correlation cofficients were significant.

	All participants (N = 60)		Without ADHD diagnosis (N = 58)	
	ADHD-I r	ADHD-II r	ADHD-I r	ADHD-II r
Face matching RT	-0.085	-0.048	-0.123	-0.070
Global form accuracy	-0.043	-0.002	-0.064	-0.017
Feature-based accuracy	-0.125	-0.016	-0.193	-0.059

Table s3. Correlation (Pearson's r) between reading measures and Behavioral Evaluation Questionnaire for Adults I and II, intended to assess symptoms of ADHD. Significant correlation coefficients are underlined in bold. Group is coded as 0: typical reader and 1: dyslexic reader.

	All participants (N = 60)		Without ADHD diagnosis (N = 58)	
	ADHD-I r	ADHD-II r	ADHD-I r	ADHD-II r
Group	**0.523**	**0.529**	**0.503**	**0.525**
ARHQ	**0.500**	**0.470**	**0.463**	**0.458**
Reading speed	**-0.254**	**-0.275**	-0.227	**-0.268**
Reading accuracy	-0.153	**-0.352**	-0.175	**-0.370**

In our original hierarchical regression analyses, we factored out variance related to ADHD by partialling out ADHD-I, ADHD-II, and ADHD diagnosis, allowing ADHD symptoms to account for all variance shared with reading problems and leaving only the variance in reading problems not accounted for by symptoms of ADHD. With the exception that only participants without a previous ADHD diagnosis are included, Table s4 is directly comparable with Table 1 in the main text.

Table s4. Summary of regression models with group membership ARHQ, reading speed and reading accuracy as dependent variables, and measures of ADHD and face processing as independent variables. Unstandardized regression coefficients (b) and p-values are bold for significant independent predictors (blue when b is positive and red when b is negative in online color version). Only participants without a previous ADHD diagnosis (N = 58) are included.

	Group		ARHQ		Reading speed		Reading acc.	
	b	*p*	*b*	*p*	*b*	*p*	*b*	*p*
Constant	11.503	0.157	0.792	0.066	-9.089	0.861	**0.627**	**<0.001**
ADHD-I	0.090	0.210	0.005	0.233	0.003	0.995	0.002	0.178
ADHD-II	**0.118**	**0.019**	0.005	0.051	-0.419	0.144	**-0.003**	**0.003**
Face matching RT	0.585	0.234	0.044	0.087	-5.748	0.065	**-0.021**	**0.020**
Global form accuracy	0.058	0.525	0.007	0.134	-0.133	0.807	0.001	0.624
Feature-based accuracy	**-0.291**	**0.019**	**-0.016**	**0.003**	**1.479**	**0.023**	**0.004**	**0.033**

S3. Reading Measures vs. Face Processing

Table s5. Zero-order correlations of reading measures with feature-based and global form face processing accuracy. No zero-order correlations were significant.

	Feature-based face accuracy	Global form face accuracy
ARHQ	-0.160	0.078
Reading speed	0.164	0.025
Reading accuracy	0.167	0.085

S4. Regression Models Accounting for Visual Search

Our data indicate that reading problems are associated with greater symptoms of ADHD. Furthermore, as detailed in the “sister” manuscript of the current primary manuscript, reading problems are also associated with slower visual search, especially conjunction search (Sigurdardottir, Omarsdottir & Valgeirsdottir, 2021). General attentional problems as indicated by symptoms of ADHD and specific problems with visual attention as captured by difficulties with visual search, particularly conjunction search, have previously been documented in the literature. Is

the association between reading problems and specific problems with feature-based face processing largely independent of their association with attentional problems – assuming that attentional problems are captured by measures of ADHD and visual search?

We attempted to answer this question by running four final regression models, summarized in supplementary table s6, with measures of ADHD, face matching, and visual search as independent variables and group membership, ARHQ, reading speed, and reading accuracy as dependent variables. Even when all other measures were factored out, feature-based face matching accuracy was a significant independent predictor of group membership, ARHQ and reading speed, while its specific association with reading accuracy was not significant. Overall, the association between task-specific problems in feature-based face matching and reading problems do not seem to be driven by attentional problems.

Table s6. Summary of regression models with group membership (logistic regression, $\chi^2(10) = 40.631$, $p < 0.001$, $R^2_{\text{Nagelkerke}} = 0.665$), ARHQ (linear regression, $F(10,49) = 4.212$, $p < 0.001$, $R^2 = 0.462$, $R^2_{adjusted} = 0.353$), reading speed (linear regression, $F(10,49) = 2.431$, $p = 0.019$, $R^2 = 0.332$, $R^2_{adjusted} = 0.195$) and reading accuracy (linear regression, $F(10,49) = 2.062$, $p = 0.046$, $R^2 = 0.296$, $R^2_{adjusted} = 0.153$) as dependent variables, and measures of ADHD, face matching, and visual search as independent variables. Unstandardized regression coefficients (*b)* and *p*-values are bold for significant independent predictors (blue when *b* is positive and red when *b* is negative). Regression coefficients for search slopes and intercepts are in seconds. It should be noted that the number of independent predictor variables is quite high (10) compared to the number of participants (60). While this could possibly pose problems for logistic regression, two subjects per variable appears to suffice to accurately estimate regression coefficients and their confidence intervals in linear regression (Austin & Steyerberg, 2015).

	Group		ARHQ		Reading speed		Reading acc.	
	b	*p*	*b*	*p*	*b*	*p*	*b*	*p*
Constant	16.199	0.145	0.491	0.332	-0.029	1.000	**0.659**	**<0.001**
ADHD-I	0.066	0.433	0.006	0.154	-0.174	0.705	0.002	0.192
ADHD-II	**0.156**	**0.017**	0.004	0.107	-0.303	0.285	**-0.002**	**0.005**
ADHD diagnosis	20.934	0.999	0.203	0.123	-10.639	0.477	0.013	0.780
Face matching RT	1.099	0.102	0.035	0.226	-5.557	0.092	-0.016	0.099
Global form accuracy	0.046	0.670	0.006	0.189	0.184	0.740	0.001	0.530
Feature-based accuracy	**-0.420**	**0.006**	**-0.014**	**0.023**	**1.341**	**0.048**	0.003	0.129
Feature intercept	**6.742**	**0.040**	0.107	0.451	-16.967	0.297	0.056	0.257
Feature slope	284.957	0.190	-0.449	0.959	1373.025	0.180	1.394	0.648
Conjunction intercept	0.290	0.634	0.025	0.467	-4.590	0.239	-0.015	0.191
Conjunction slope	-27.171	0.223	0.225	0.819	30.222	0.789	-0.023	0.946

Supplementary References

Austin, P. C., & Steyerberg, E. W. (2015). The number of subjects per variable required in linear regression analyses. *Journal of Clinical Epidemiology, 68*(6), 627-636.

Brady, N., Darmody, K., Newell, F., & Cooney, S. M. (2020, March 4). Holistic Processing of Faces and Words Predicts Reading Accuracy and Speed in Dyslexic Readers. *PsyArXiv*. https://doi.org/10.31234/osf.io/abys2

Baker, C. I., Behrmann, M., & Olson, C. R. (2002). Impact of learning on representation of parts and wholes in monkey inferotemporal cortex. *Nature Neuroscience, 5*(11), 1210-1216.

Brooks, D., Sigurdardottir, H. M., & Sheinberg, D. L. (2014). The neurophysiology of attention and object recognition in visual scenes in. In K. Kveraga & M. Bar (Eds.), *Scene Vision* (pp. 85-104). Cambridge, MA: MIT Press.

Driver, J., Davis, G., Ricciardelli, P., Kidd, P., Maxwell, E., & Baron-Cohen, S. (1999). Gaze perception triggers reflexive visuospatial orienting. *Visual Cognition, 6*(5), 509-540.

Fairweather, H., Brizzolara, D., Tabossi, P., & Umiltà, C. (1982). Functional cerebral lateralisation: Dichotomy or plurality? *Cortex, 18*(1), 51-65.

Friesen, C. K., & Kingstone, A. (1998). The eyes have it! Reflexive orienting is triggered by nonpredictive gaze. *Psychonomic Bulletin & Review, 5*(3), 490-495.

Hemond, C. C., Kanwisher, N. G., & De Beeck, H. P. O. (2007). A preference for contralateral stimuli in human object-and face-selective cortex. *PLOS ONE, 2*(6), e574.

Hillger, L. A., & Koenig, O. (1991). Separable mechanisms in face processing: Evidence from hemispheric specialization. *Journal of Cognitive Neuroscience, 3*(1), 42-58.

Kay, K. N., Weiner, K. S., & Grill-Spector, K. (2015). Attention reduces spatial uncertainty in human ventral temporal cortex. *Current Biology, 25*(5), 595-600.

Lachmann, T. (2018). Reading and dyslexia: The functional coordination framework. In *Reading and Dyslexia* (pp. 271-296). Springer, Cham.

Pitcher, D., Pilkington, A., Rauth, L., Baker, C., Kravitz, D. J., & Ungerleider, L. G. (2019). The human posterior superior temporal sulcus samples visual space differently from other face-selective regions. *Cerebral Cortex*. Advance online publication: doi: 10.1093/cercor/bhz125

Sergent, J. (1982). About face: Left-hemisphere involvement in processing physiognomies. *Journal of Experimental Psychology: Human Perception and Performance, 8*(1), 1-14.

Sigurdardottir, H. M., & Jozranjbar, B. (2019). Laterality effect (face perception). In J. Vonk & T. Shackelford (Eds.), *Encyclopedia of Animal Cognition and Behavior*. New York: Springer.

Sigurdardottir, H. M., Omarsdottir, H. R., & Valgeirsdottir, A. S. (2021, March 25). Reading problems and their connection with visual search and attention: The search for a cause continues. *PsyArXiv*. https://doi.org/10.31234/osf.io/5j9ys

Sigurdardottir, H. M., Michalak, S. M., & Sheinberg, D. L. (2014). Shape beyond recognition: Form-derived directionality and its effects on visual attention and motion perception. *Journal of Experimental Psychology: General, 143*(1), 434-454.
